# Extract from a Letter to the Surgeon General of Massachusetts

**Published:** 1863-05

**Authors:** J. F. Galloupe

**Affiliations:** Surg. 17th Mass. Vols.


### CHICAGO MEDICAL JOURNAL.

VOL. VI.] MAY, 1863. [No. 5.

### SELECTED.

### EXTRACT FROM A LETTER TO THE SURGEON GENERAL OF MASSACHUSETTS.

From J. F. GALLOUPE, M. D., Surg. 17th Mass. Vols.

I presume that before this time you have had full details of the "malarial disease," as it is called, which now prevails here; the disease is not malarial in its origin. The fact that it prevails in the winter, when malarious disease does not originate; that it prevails among troops recently arrived here; that it is not paroxysmal in its character; that it is not modified by quinine; and that the pathological changes are totally unlike those which occur in paludal disease, all prove that it is not due to miasm.

I. *Cause.*—That the disease is caused *generally* by living in barracks made of green lumber, and insufficient ventilation, is obvious. The 43d, 45th and 51st regiments were encamped as near together as it was possible for them to be. The former was in tents, and had none of the disease. The two latter were in barracks, and in about four weeks after their arrival the disease began to appear. Upon the average, two cases and two deaths per week occurred in each of these regiments. At last the prevalence of the disease created alarm, and the 45th were ordered to change their quarters; this being done, no new cases appeared. The disease continued to prevail in the 51st until they were removed from barracks, when it suddenly disappeared. What has been said of the 45th and 51st, is also true of the 44th.

The barracks are made of perfectly green hard-pine lumber, and so constructed as to allow only 180 feet of air to each man, with no adequate ventilation. During the stay of the 45th in barracks they had five cases, all of which were fatal. The 51st have had seventeen cases, all of which have been fatal. The 44th have had twenty cases, twelve of which have been fatal; the remaining eight are now under treatment. One case of this disease occurred in my regiment last summer, in a man who was employed in building a block-house of green timber; he occupied the building as his quarters.

I have said that the disease is *generally* caused by living in barracks made of green lumber; but this is not the only cause, for a few cases have occurred among troops not thus quartered. A lad nine years old, son of one of our officers, was attacked with the disease; he lived in a good brick house, and was in all respects well cared for, and, up to the time of the attack, had been perfectly healthy. His case exactly resembled those which occurred among the troops—in fact it was a *pattern* case.

The victims of this disease, so far as my observation extends, have been the most healthy and vigorous men. I have conversed with several of the most intelligent people who reside here (including a physician), and they all agree in the statement that the disease, as an epidemic, never existed here before.

II. *Symptoms.*—The patient is attacked, without warning, with a rigor more or less severe; pain in the head, back and legs, the latter being the most severe; delirium; vomiting; thick white coat on the tongue; bowels constipated; sometimes retention of urine. In some cases "petechiæ" of large size are extremely plentiful from the *outset*, in other cases this symptom does not appear. In some cases the force of the disease is so great as to destroy life within twelve hours; in others, the patient continues to live without marked change in the symptoms until the strength gradually succumbs, in a period varying from two days to several weeks. I have not yet known a case of perfect recovery; the one nearest approaching it, that I have seen, was a case which came before the Board of Examiners for discharges, of which I am a member; this man was completely broken down; he had chronic iritis and permanent blindness of one eye.

III. *Pathology.*—Uniformly the disease is found to be inflammation of the meninges of the brain and spinal cord. When death occurs early, say in twelve hours after the attack, the arachnoid at the top of the brain is found to be opaque; the disease extends from this point to the base of the brain, and down the spinal cord. If the patient survives the attack for two or three days, large coagula of lymph are found beneath the dura mater, from the top of the brain to the cauda equina; at this stage of the disease the lymph is of a greenish-yellow color, and partially disorganized; there is also some softening of the cerebral substance, but I have not yet seen pus. Sometimes the dura mater is adherent. There is also effusion of serum beneath the dura mater, and into the ventricles, amounting in some cases to five ounces. In many cases a considerable amount of congestion is found, but this condition is by no means constant. The disease appears to originate in the arachnoid. In all the cases that I have seen no other organic lesion has been found, acute or chronic, than the one above described, although the thoracic and abdominal viscera have been carefully examined.

IV. *Treatment.*—No treatment has been of any avail. When the disease first appeared it was supposed by those who had it in charge to be of malarious origin, and was consequently treated by quinine. Other cases have been treated antiphlogistically—by venesection, cupping, blisters, cold applications, sinapisms to extremities, cathartics and mercurials. When petechiæ appeared early in the disease it was treated as typhus, with oil of turpentine, &c., and supporting diet. These different plans of treatment have been combined as indicated by the symptoms, and, so far as my knowledge extends, without favorably modifying the course of the disease.—*Ibid.*

